# Alleviation of acute stress response by black pepper aroma administration

**DOI:** 10.1186/s40101-023-00352-1

**Published:** 2024-01-02

**Authors:** E. A. Chayani Dilrukshi, Yuta Nishiyama, Kanetoshi Ito, Shusaku Nomura

**Affiliations:** 1 Graduate School of Engineering, Nagaoka University of Technology, Nagaoka, Niigata, 940-2188 Japan; 2 Department of Industrial Management, Faculty of Applied Sciences, Wayamba University of Sri Lanka, Kuliyapitiya, 60200 Sri Lanka; 3 Corporate Research & Development, Takasago International Corporation, Hiratsuka, Kanagawa 254-0073 Japan

**Keywords:** Black pepper, Heart rate, Heart rate variability, Skin conductance level, Acute stress response, Autonomic nervous system activity

## Abstract

**Background:**

Black pepper is one of the most popular spices globally. As black pepper essential oil has not yet been used in the context of aromatherapy, this study examined the effect of black pepper aroma on cardiac and peripheral autonomic nervous system (ANS) activity under stressful conditions using an olfactometer to administer aroma in a precise and controlled manner to ensure reproducibility.

**Methods:**

A within-participant design experiment was conducted with 20 male university students who performed a 30-min calculation task as a short-term stressor under three aroma conditions: black pepper, ginger, and dipropylene glycol (DPG) (scentless air as a control). Each aroma was sporadically delivered (first 20 s of each 1-min interval) with the olfactometer during the task. Electrocardiograms and skin conductance level (SCL) were measured to evaluate ANS's physiological acute stress response. Subjective evaluations for the given stressful task and impressions on the types of aromas were assessed.

**Results:**

The physiological acute stress response induced by the short-term stressor, which is characterized by the enhancement of the heart rate (HR) and SCL and decreases in the heart rate variability (HRV), was suppressed with black pepper: the increase in HR and reduction in HRV from the baseline were 38.9% (*p* = 0.048 when compared with DPG) and 32.9% smaller (*p* = 0.002 for multiple comparisons) than those in DPG, respectively, and the increase of SCL was 15.5% smaller (*p* = 0.005 for multiple comparisons) than that in ginger. However, there was no significant difference in subjective scores among the conditions.

**Conclusion:**

Although black pepper is a stimulative agent, the study findings showed that black pepper aroma alleviated the physiological acute stress response, which can be beneficial in aromatherapy under stressful conditions.

## Background

Aromatherapy has gained popularity globally in complementary and alternative medicine. It uses different types of essential oils, and various psychological and physiological effects are expected from these aromas. Previous studies on aromatherapy have reported reduced anxiety with lavender [1, 2], orange [3–6], sandalwood [7], citrus ginger [8], Hiba [1], and *Melissa officinalis* (lemon balm) extract [9]; relieved stress with lemon [10], peppermint [11], black tea [12], green tea [13] and blended essential oil with sandalwood and lavender [14]; improved sleep quality with lavender [2, 15, 16] and *Rosa damascene* [17]; induced physiological relaxation with Hinoki cypress leaf oil [18], and increased memory performance with lavender, rosemary [19], and ginger [20].

Ginger (*Zingiber officinale* Roscoe), a popular edible spice, has also been reported in previous studies to show various psychophysiological benefits and pain relief. Inhalation of ginger essential oil has demonstrated positive effects on nausea and vomiting severities [21–23] and ginger oil massage has been effective in relieving chronic low back pain [24] and moderate-to-severe knee pain [25] in older adults, while ginger extract showed positive efficacy in pain relief and improved functionality in patients with moderate knee osteoarthritis [26]. Moreover, inhalation of citrus ginger during sleep enhanced sympathetic nervous system (SNS) activity upon awakening and alleviated psychological tension and anxiety [8].

In black pepper (*Piper nigrum* L*.*), known as the ‘king of spices’, the flavor comes from the compound piperine [27]. Black pepper has functional uses in culinary, perfumery, and traditional medicine [28, 29]. Few studies have assessed the efficacy of its essential oils in medical use, and an aromatic essential oil cream comprising black pepper, marjoram, lavender, and peppermint has been reported to improve pain tolerance and neck pain [30]. Additionally, studies have suggested that inhaled black pepper oil may improve postural stability in older adults by reducing the velocities of posture adjustments [31] and the sensory and reflexive motor movement of swallowing via activation of the right insular cortex in patients with dysphagia [32, 33]. Two studies reported the effect of topically applied black pepper essential oil for enhancing vein visibility in patients, thus aiding easy intravenous catheter insertion [34, 35].

In contrast to previous studies, which primarily evaluated the stimulant properties of black pepper aroma for specific clinical outcomes, this research delves into unexplored research areas by investigating the therapeutic potential of black pepper essential oil within the context of aromatherapy. Notably, this study extends beyond assessing arousal efficiency to comprehensively explore therapeutic benefits, considering the stimulative sensation of black pepper as a food ingredient and its positive efficacies. These positive effects include pain relief and its potent warming effect [36], similar to other stimulative spices such as mustard and ginger, which are thought to increase blood flow, attributing to attenuation of peripheral SNS activity [37, 38].

Focusing on the therapeutic efficiency of black pepper essential oil, we employed a psychological loading test methodology in this study. In studies assessing physiological stress responses to short-term stressors, electrocardiograms (ECG) and skin conductance levels (SCL) are frequently used as physiological measures. Thoma et al. (2013) observed a subsequent change in heart rate (HR) while listening to music in a stressful situation [39]. Enhanced parasympathetic nervous system (PSNS) activity indicated by the high frequency (HF) component of heart rate variability (HRV) was observed with Zen meditation [40], soothing music [41], and forest bathing [42]. Additionally, research on using citrus mint-flavored mouthwash revealed a significant reduction in SCL during the recovery period following a calculation task [43]. Hence, employing physiological measures such as ECG and SCL to evaluate the acute stress response may yield promising results.

Controlling the concentration and duration of aroma administration has been a challenge in many previous studies, and the outcomes of those studies might have been affected by olfactory fatigue. Most of those studies adopted conventional methods for administering aroma, including aroma diffusers [44–46] or impregnating materials with aroma [4]. Therefore, to prevent olfactory fatigue, this study used a customized olfactometer, which precisely controlled the concentration and duration of aroma inhalation to ensure experiment reproducibility.

Thus, this study aimed to investigate the alleviating efficacy of black pepper in aromatherapy under stressful conditions by determining the acute physiological stress responses in the autonomic nervous system (ANS) in a well-controlled laboratory setting using an olfactometer. We hypothesized that black pepper aroma would alleviate physiological stress responses to a short-term cognitive stressor, specifically suppressing the increase in HR and SCL and the decrease in HF compared to the scentless condition.

## Methods

### Participants

The study comprised 20 healthy male university students with a mean ± standard deviation (SD) age of 21.5 ± 0.87 years (the range of age is from 24 to 20 years) and a mean ± SD body mass index (BMI) of 22.8 ± 3.2 kg/m^2^ (the range of BMI is form 18.2 kg/m^2^ to 29.1 kg/m^2^). None of the participants have any previous record of illnesses related to olfactory function. Pre-screening was conducted before the experiment for each participant to check the normality of their sense of odor using a simple olfactory testing kit (Open Essence, FUJIFILM Wako Pure Chemical Corporation, Japan).

The study was conducted following the ethical principles of the Helsinki Declaration of 1975, as revised in 2000 (5), and informed consent was obtained from all participants. The study design was approved by the ethics committee of the Nagaoka University of Technology.

### Experimental procedure

#### Experimental protocol

**Fig. 1:**
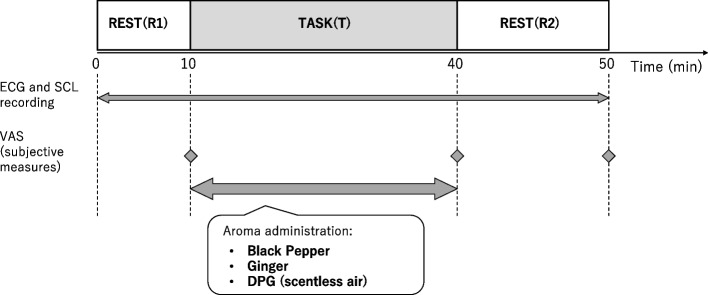
Schematic presentation of the experimental protocol. Participants performed a 30-min calculation task as a short-term stressor under three aroma conditions: black pepper, ginger, and dipropylene glycol (DPG) (scentless air as a control). Each participant experienced three aroma conditions on three separate days (within-participant design). The order of aroma conditions was counterbalanced

Figure 1 shows the experimental protocol. Each participant was instructed to rest for 10 min (R1), perform a 30-min calculation task to induce cognitive stress (T), and undergo a 15-min recovery period (R2). The Kraepelin test, commonly conducted in laboratory studies, was used as the cognitive stressor in this study [5, 47]. It is performed by displaying a single one-digit addition exercise on a computer screen for 30 min. Although the task was simple, it entailed a high degree of focus and concentration. Furthermore, the participants were guided to complete the task as quickly and precisely as possible. Each participant experienced three aroma conditions, including black pepper, ginger, and scentless air, on three separate days. The order of aroma conditions was counterbalanced. All experiments were conducted in an air-controlled laboratory with a mean (± SD) temperature of 25.1 ± 0.8 °C and humidity of 58.9% ± 6.4%.

#### Aroma essential oils

Black pepper essential oil (Takasago International Corporation, Kanagawa, Japan) was used as the main component, and ginger essential oil (Takasago International Corporation, Kanagawa, Japan) was employed as a typical “stimulant” and a reference aroma. Major constituents in black pepper and ginger essential oils are given in Table 1. A scentless solvent, dipropylene glycol (DPG), was used as the control stimulus in the study.

**Table 1:** Major constituents of essential oils used in the study

Essential oil	Constituent	(%)
Black pepper	β-Caryophyllene	35.1
	Limonene	15.0
	α-Pinene	10.3
	β-Pinene	10.2
	δ-3-Carene	8.5
Ginger	α-Zingiberene	36.5
	β-Sesquiphellandrene	14.1
	(3E,6E)-α-Farnesene	8.1
	β-Bisabolene	7.3
	α-Curcumene	5.6

#### Aroma administration

Black pepper, ginger aroma, or DPG was sporadically administered (first 20 s of each 1-min interval) through a cannula placed under the nostrils and connected to a custom-built olfactometer (Tatsumi Kagaku Co. Ltd, Kanazawa, Japan). The scentless air flowed into the aroma at a rate of 1.5 L/min to volatilize the essential oil. The volatilized essential oil was conveyed to the cannula by regulating the timing and volume using an electromagnetic valve, which was precisely controlled on the order of microseconds following the pre-programmed timing. This timing was programmed through our developed interface on the personal computer to achieve sporadic delivery of the aroma. Olfactory fatigue of participants was prevented by the sporadic delivery of aromas [5, 47].

### Measurements

#### Physiological measurements

During the experiment (R1-T-R2), an ECG was recorded using a bio-amplifier (MP150, BIOPAC Systems Inc., CA) at a sampling rate of 500 Hz and a resolution of 16-bit to analyze the HR and HRV. Electrodes were placed underneath the right clavicle and on the lower left abdomen following the Lead II placement protocol for ECG measurements (ECG sensor: TSD155C, BIOPAC Systems Inc.). After the acquisition, the signals were filtered using a 35 Hz low-pass filter, a 50 Hz notch filter, and a 0.05 Hz low-cut filter. The HF component (range 0.15–0.40 Hz) of HRV was identified as a measure of cardiac PSNS activity [18, 48, 49]. Additionally, the SCL, as a measure of SNS, was recorded using the same bio-amplifier at a sampling rate of 500 Hz with 16-bit resolution. The sensors were placed on the palmar side of the middle phalanx of the index and ring fingers of the participant’s non-dominant hand (SCL sensor: TSD203, BIOPAC Systems Inc.). A low-pass filter at 1.0 Hz was used after the signal acquisition. All analyses were performed using the software (AcqKnowledge 4.1, BIOPAC Systems Inc.) dedicated to the bio-amplifier.

#### Psychological measurements

Subjective measures were assessed using the visual analog scale (VAS) and a scent questionnaire, as commonly used in previous studies [5, 50]. VAS, a calibrated line with two endpoints of 0 and 100% [12], comprising seven items; ‘Nervousness’, ‘Effort’, ‘Concentration’, ‘Fatigue’, ‘Irritation, ‘Boredom’, and ‘Fed-up’ were given to the participants at the end of R1, T, and R2 (Fig. 1). The participants rated their perception of each of the seven items on their respective points in less than 10 s. Furthermore, a scent questionnaire was provided to rate the ‘Comfort’, ‘Strength’, ‘Preference’, and ‘Awakening’ of each aroma condition using a 7-point Likert scale (1–7; strongly disagree–strongly agree) at the end of T.

### Statistical analysis

The raw values of HR, HF, and SCL were standardized (*z*-score) considering each participant and condition, owing to the significant individual variations, and all the values were baseline-corrected regarding the mean value at R1. After conducting the Shapiro-Wilk test, paired *t* tests were performed to compare subjective and objective parameters within and among the conditions if the datasets could be considered normally distributed; otherwise, Wilcoxon signed-rank tests were performed as well. Bonferroni corrections were used for multiple comparisons among the conditions. Even if no significant difference was found in multiple comparisons, if the statistical test results “before” applying the Bonferroni correction (i.e., results of the pairwise test) had a *p* value < 0.05, it was noted as informative information. To explore the relationship between subjective impressions of aroma and physiological measures, Spearman’s rank correlation coefficient was determined. The statistical significance level for *p* was set at 0.05. Statistical analysis was performed using R software (version 4.2.2., R Foundation for Statistical Computing, Vienna, Austria).

## Results

### Heart rate and heart rate variability

**Fig. 2:**
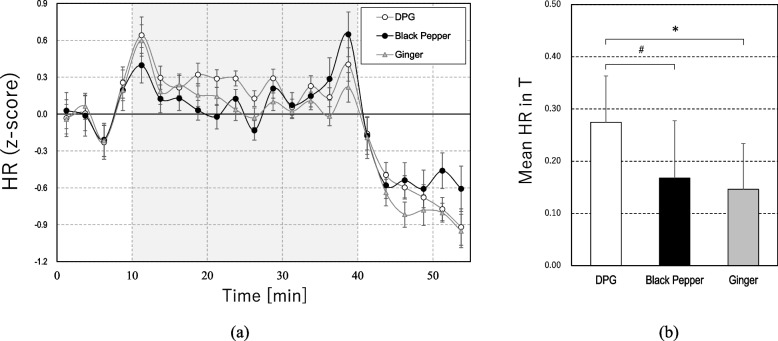
**a** Changes in heart rate (HR) (mean ± SEM per 2.5 min) and **b** mean HR value during the task (T) (mean ± SEM), *p < 0.05, ^#^*p* < 0.05 before multiple comparisons

**Fig. 3:**
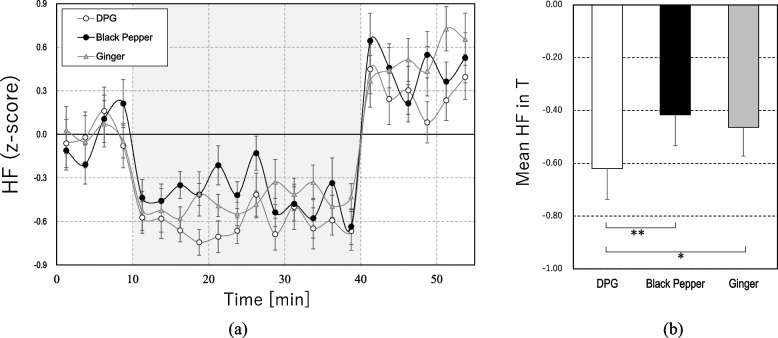
**a** Changes in high-frequency (HF) component of heart rate variability (HRV) (mean ± SEM per 2.5 min) and **b** mean HF component value during the task (T) (mean ± SEM), *p < 0.05, **p < 0.01

Figures 2a and 3a show the change in HR and HF components of HRV for the three conditions. The results showed an enhancement of HR and a decrease in HF during the task (T) compared to R1 (*ps* < 0.01) regardless of the conditions, which indicated a typical acute stress response of the SNS and PSNS [47, 51].

Figures 2b and 3b illustrate the mean HR and HF values during the task. The results showed that black pepper and ginger maintained lower levels in HR than in DPG and relatively higher levels in HF than in DPG. The acute stress-induced enhancement in HR during the task was marginally smaller in black pepper (38.9% smaller than that in DPG, *t*_19_ = 2.11, *p* = 0.048; note that it was determined by a paired comparison with DPG) and was significantly smaller in ginger (46.8% smaller than that in DPG, *t*_19_ = 2.89, *p* = 0.028) than in DPG. The acute stress-induced decrease in HF during the task was significantly smaller with black pepper (32.9% smaller than that in DPG, *t*_19_ = 4.04, *p* = 0.002) and ginger (25.2% smaller than that in DPG, *t*_19_ = 2.82, *p* = 0.033) than with DPG. The smaller enhancement of HR and decrease in HF by black pepper and ginger compared to DPG indicated the suppression of acute stress response on cardiac SNS and PSNS by black pepper and ginger.

### Skin conductance level

**Fig. 4:**
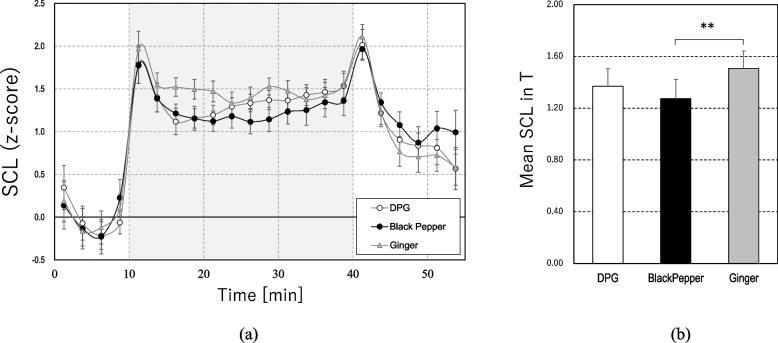
**a** Changes in skin conductance level (SCL) (mean ± SEM per 2.5 min) and **b** mean value of SCL during task (T) (mean ± SEM), **p < 0.01

Figure 4a shows the changes in the SCL. For all three conditions, SCL was enhanced during the task compared with R1 (*ps* < 0.001), which represented a typical acute stress response of peripheral SNS [50, 51]. According to the mean SCL values during the cognitive task, as shown in Fig. 4b, the enhancement in SCL during the task in black pepper was significantly smaller than in ginger (15.5% smaller than that in ginger, *t*_19_ = 3.63, *p* = 0.005). The SCL value of black pepper remained at the lowest level through most of the T as shown in Fig. 4a and was lower in mean value than that of DPG, while it did not reach a significant level, representing a suppression of the peripheral SNS elevation by black pepper.

### Subjective impressions and VAS scores

Table 2 demonstrates the subjective impressions of each aroma. All participants in this study could distinguish between the three conditions used: DPG, black pepper, and ginger. According to these subjective impressions, black pepper and ginger were stronger than DPG (*ps* < 0.001). There were no significant differences between the conditions for the other items.

**Table 2:** Results of subjective impressions for each aroma condition (7-point Likert scale)

	Condition	Mean	(SD)
Comfort	DPG	4.25	(1.02)
	BLP	3.60	(1.39)
	GIN	4.20	(1.24)
Strength	DPG	2.50 ^†, ‡^	(1.40)
	BLP	4.45 ^†^	(0.94)
	GIN	3.90 ^‡^	(0.79)
Preference	DPG	4.25	(1.12)
	BLP	3.55	(1.19)
	GIN	4.10	(1.17)
Awakening	DPG	3.90	(1.02)
	BLP	4.80	(1.32)
	GIN	3.90	(1.21)

*DPG* dipropylene glycol, *BLP* black pepper, *GIN* ginger, *SD* standard deviation

^†, ‡^*p* < 0.001, Bonferroni-corrected comparison between each condition

**Fig. 5:**
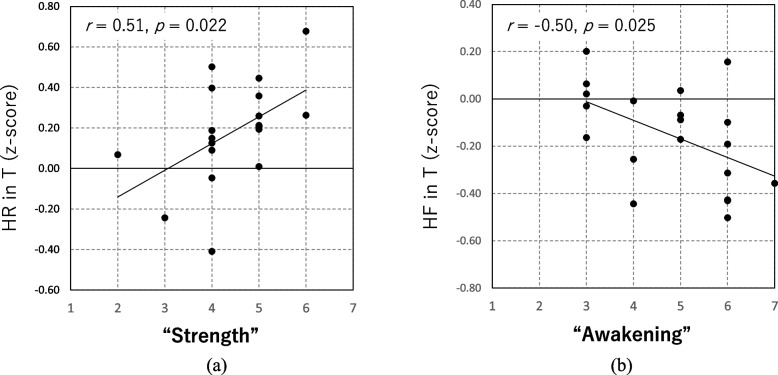
**a** Correlations observed between ‘Strength’ and mean HR during the task and **b** Correlations observed between ‘Awakening’ and mean HF during the task

In addition, significant positive and negative correlations were observed between ‘Strength’ and mean HR during the task (*n* = 20, *r* = 0.51, *p* = 0.023) and between ‘Awakening’ and mean HF during the task (*n* = 20, *r* = − 0.50, *p* = 0.025) in black pepper as shown in Fig. 5a, b respectively. There was no correlation between ‘Preference’ and ‘Comfort’ and any of the physiological measures for all aroma conditions.

Table 3 shows the VAS scores for R1, T, and R2. Regardless of the conditions, VAS scores indicated an increase in ‘Nervousness’, ‘Effort’, ‘Concentration’, ‘Fatigue’, ‘Irritation’, and ‘Fed-up’ and a decrease in ‘Boredom’ during T compared to at R1. Therefore, the 30-min calculation task employed in the study functioned as a short-term cognitive stressor similar to a previous study [47]; It should be noted that, similar to other studies [5, 47], there was no significant difference in task performance observed between the aroma conditions. The scores for ‘Fed-up’ in black pepper were significantly lower than those in DPG during T (*t*_19_ = 2.63, *p* = 0.049). However, there were no significant differences between the three conditions in each period.

**Table 3:** Results of subjective measures (VAS scores) for each aroma condition

VAS score	Condition	Rest (R1)		Task (T)		Rest (R2)	
		Mean	(SD)	Mean	(SD)	Mean	(SD)
Nervousness	DPG	31.2	(22.6)	32.4	(19.7)	25.5	(18.4)
	BLP	24.0	(17.1)	41.4	(20.1)	23.4	(19.0)
	GIN	24.1	(19.4)	35.0	(19.9)	20.3	(16.6)
Effort	DPG	30.8	(20.4)	50.7	(22.7)	26.9	(20.6)
	BLP	25.7	(19.5)	49.9	(20.7)	24.7	(21.7)
	GIN	25.5	(21.3)	49.8	(23.0)	22.8	(17.7)
Concentration	DPG	40.6	(22.7)	50.1	(22.9)	31.8	(21.0)
	BLP	38.0	(22.7)	53.4	(24.8)	29.3	(21.4)
	GIN	35.2	(23.9)	45.8	(24.9)	30.5	(24.6)
Fatigue	DPG	28.2	(21.3)	51.0	(23.7)	39.6	(23.5)
	BLP	22.4	(18.8)	49.0	(21.0)	41.8	(22.1)
	GIN	25.8	(20.2)	51.2	(21.6)	35.8	(18.1)
Irritation	DPG	20.2	(19.5)	30.1	(21.1)	22.2	(17.9)
	BLP	17.4	(17.0)	26.6	(17.6)	22.8	(18.1)
	GIN	18.9	(16.7)	34.2	(20.2)	17.9	(15.8)
Boredom	DPG	39.0	(21.4)	28.3	(20.6)	43.2	(20.7)
	BLP	39.1	(21.3)	25.7	(19.6)	43.5	(19.1)
	GIN	42.3	(19.7)	27.0	(18.2)	41.6	(20.3)
Fed-up	DPG	25.1	(21.7)	36.7 ^*^	(20.3)	30.2	(23.7)
	BLP	23.4	(15.1)	30.0 ^*^	(17.4)	30.3	(18.5)
	GIN	25.4	(17.9)	38.3	(22.1)	28.7	(18.0)

*DPG* dipropylene glycol, *BLP* black pepper, *GIN* ginger, *VAS* visual analog scale, *SD* standard deviation; ^*^ *p* = 0.049, Bonferroni-corrected comparison between each condition

## Discussion

This study evaluated the effects of black pepper aroma on peripheral and cardiac ANS activity under stressful conditions using a customized olfactometer to ensure subsequent reproducibility. As for the results, the acute stress response in HF was suppressed in black pepper and ginger compared with DPG, and that in SCL was suppressed in black pepper compared with ginger. There was a marginal suppression of acute stress by black pepper compared with DPG concerning HR. Regarding the subjective impressions of the participants, black pepper and ginger were stronger than DPG, while there was no significant difference in psychological parameters between the three conditions for the other items. Although black pepper has been used as a preservative and stimulative spice, our findings suggested that it has an alleviative effect under stressful conditions.

The biological mechanism underlying these effects of black pepper aroma inhalation on ANS response remains unclear. β-caryophyllene, the main component contained in the black pepper essential oil used in the study, is found in many dietary condiments, including cloves, oregano, thyme, and cinnamon [52]. Although animal studies have shown that β-caryophyllene may attenuate hyperglycemia [53], its physiological effects have not been extensively studied. The pharmacological effects of α- and β-pinene isomers have been extensively studied. Previous research has shown they have critical pharmacological effects in animals, including antibacterial, antioxidant, and anti-inflammatory effects [54, 55]. However, the lack of research on human subjects makes it difficult to discuss physiological background concerning the results of psychophysiological studies such as the present study. On the other hand, one study on olfactory stimulation with limonene demonstrated enhanced HF and significantly lower HR in relaxing situations [56], providing some insights into the physiological effects of limonene. These effects may lead to the current study findings.

It is difficult to compare this study with previous studies directly. This is because, to the best of our knowledge, only a few studies have been conducted on black pepper aroma. Although black pepper is a frequently utilized dietary condiment globally, several past studies have investigated black pepper in the context of pain relief. Ou et al. 2014 assessed the efficacy of an aromatic essential oil cream composed of black pepper, marjoram, lavender, and peppermint (2:2:1:1) on neck pain and reported improved pain tolerance and significant improvement in neck pain. SNS activity is associated with the generation of pain [57, 58], and the reduction of pain using aromatic essential oil cream can be suggested as a suppression of SNS activity. The suppression of the cardiac and peripheral SNS response by black pepper observed in our study is consistent with these findings. Furthermore, there was no significant difference in the subjective impressions between the conditions; this demonstrated the physiological effects of black pepper, with no significant difference in psychological effects.

Generally, black pepper is believed to act as a stimulant during consumption because of its spicy sensation, and this stimulatory effect has been observed in several previous studies. The inhalation of black pepper oil resulted in the improvement of reflexive swallowing movement in older patients with dysphagia, which would help prevent aspiration pneumonia [32, 33]. Black pepper aroma used in smoking cessation programs stimulates respiratory tract sensations, thereby reducing the level of craving for nicotine and cigarettes [59]. The activation of SNS can widen the bronchial passages and provide support for inhalation, while PSNS stimulates swallowing and digestion [36]. The stimulative effect of black pepper has been shown to affect the ANS response of the body in behavioral and clinical studies.

A positive correlation was observed between ‘Strength’ and HR and a negative correlation between ‘Awakening’ and HF in black pepper. This indicates a reasonable relationship between psychological state and physiological state as ‘Strength’ and ‘Awakening’ connect to ‘Stimulative’ sensation in some sense. Specifically, the more a participant sensed black pepper as ‘Strong’, HR increased, and the more a participant sensed it as ‘Awakening’, HF was suppressed. In this sense, the black pepper remained to be perceived as a stimulant. Thus, black pepper should have both psychological and physiological effects (at least stronger than those of ginger or DPG), and their relationship seemed reasonable (such as SNS activation followed by stimulative sensation). However, in the context of stressful situations, the physiological alleviative effect demonstrated in our study was remarkable and effective. Some studies have investigated the health benefits of black pepper as a food ingredient and stimulative. In contrast, few studies have identified the psychophysiological benefits of black pepper aroma inhalation. To the best of our knowledge, there has not been a study that demonstrated the positive efficacy of black pepper essential oil under stressful situations. As black pepper is a readily available spice worldwide, the essential oil can be consumed as a handy spice in the kitchen. Although some people may prefer not to use it in their food, it can be beneficial in aromatherapy.

One of the limitations of this study was the inclusion of only male university students. The age range was narrow, 4 years, and those classified as obese by WHO criteria (BMI ≥ 30) [60] were not included. Including such a homogeneous study population was intentional to avoid discrepancies in the results. There are significant differences in human olfactory abilities with age [61, 62] and women generally outperform men in olfactory abilities [63]. Female hormonal imbalance during specific phases of the menstrual cycle can affect olfaction and may result in variations in SNS and PSNS activity [64–66]. Subjective impressions, including the preference for aroma, also vary depending on factors such as sex, age, ethnicity, or cultural exposure to specific odors [67, 68]. Even though the study used a homogeneous sample to exclude possible interferences, there is room for future research by introducing a heterogeneous study population, which would enable the exploration of the differences between subgroups within the population. The Kraepelin test is a widely used stressor in the laboratory setting [47]. However, employing various stressors other than the Kraepelin test may be a better solution to avoid substantial limitations. The study faced technical limitations in precisely determining aroma concentration; it is a technical constraint common to aroma research. These challenges should be noted when interpreting the study results. This study did not assess the effects of black pepper when it is consumed. Hence, future research should focus on the systematic administration of black pepper essential oil in a similar or different experimental background to assist in further exploration of the benefits of black pepper aroma.

## Conclusion

This study investigated the psychophysiological effects of black pepper aroma on peripheral and cardiac ANS activities under short-term cognitive stress. The experimental results suggested that inhalation of black pepper aroma suppressed the acute stress response in the ANS. Although black pepper is a stimulative agent, the study findings suggested that it can function as an alleviative agent in stressful situations. Since black pepper is one of the most popular spices in the world, black pepper essential oil can be used in aromatherapy to alleviate stress under stressful conditions.

## Data Availability

The datasets supporting the conclusions of this article are available in the OSF repository, https://osf.io/bu5e3/?view_only=cdf4ffc558a344b39d44d79de94df1ae.

## Abbreviations

ANS: Autonomic nervous system
DPG: Dipropylene glycol
SCL: Skin conductance level
HR: Heart rate
HF: High frequency
HRV: Heart rate variability
PSNS: Parasympathetic nervous system
SNS: Sympathetic nervous system
SD: Standard deviation
